# Clinical and Molecular Characterization of Patients with Mucopolysaccharidosis Type I in an Algerian Series

**DOI:** 10.3390/ijms17050743

**Published:** 2016-05-17

**Authors:** Abdellah Tebani, Lahouaria Zanoutene-Cheriet, Zoubir Adjtoutah, Lenaig Abily-Donval, Carole Brasse-Lagnel, Annie Laquerrière, Stephane Marret, Abla Chalabi Benabdellah, Soumeya Bekri

**Affiliations:** 1 Department of Metabolic Biochemistry, Rouen University Hospital, Rouen 76031, France; abdellah.tebani@chu-rouen.fr (A.T.); zoubir.adjtoutah@jsbio.fr (Z.A.); carole.lagnel@univ-rouen.fr (C.B.-L); 2 Region-Inserm Team NeoVasc ERI28, Laboratory of Microvascular Endothelium and Neonatal Brain Lesions, Institute of Research for Innovation in Biomedicine, Normandy University, Rouen 76031, France; lenaig.abily-donval@chu-rouen.fr (L.A.-D.); annie.laquerriere@chu-rouen.fr (A.L.); stephane.marret@chu-rouen.fr (S.M.); 3 Department of Pediatrics, Oran University Hospital, Oran 31000, Algeria; houaria26dz@yahoo.fr (L.Z.-C.); ab_chalabi@yahoo.fr (A.C.B.); 4 Department of Neonatal Pediatrics and Intensive Care, Rouen University Hospital, Rouen 76031, France; 5 Pathology Laboratory, Rouen University Hospital, Rouen 76031, France

**Keywords:** mucopolysaccharidosis type I, *IDUA*, GAGs, heparin sulfate, dermatan sulfate

## Abstract

Mucopolysaccharidoses (MPS’s) represent a subgroup of lysosomal storage diseases related to a deficiency of enzymes that catalyze glycosaminoglycans degradation. Mucopolysaccharidosis type I (MPS I) is a rare autosomal recessive disorder caused by a deficiency of α-l-iduronidase encoded by the *IDUA* gene. Partially degraded heparan sulfate and dermatan sulfate accumulate progressively and lead to multiorgan dysfunction and damage. The aim of this study is to describe the clinical, biochemical, and molecular characteristics of 13 Algerian patients from 11 distinct families. MPS I diagnosis was confirmed by molecular study of the patients’ *IDUA* gene. Clinical features at the diagnosis and during the follow-up are reported. Eighty-four percent of the studied patients presented with a mild clinical phenotype. Molecular study of the *IDUA* gene allowed the characterization of four pathological variations at the homozygous or compound heterozygote status: *IDUA* NM_00203.4:c.1598C>G-p.(Pro533Arg) in 21/26 alleles, *IDUA* NM_00203.4:c.532G>A-p.(Glu178Lys) in 2/26 alleles, *IDUA* NM_00203.4:c.501C>G-p.(Tyr167*) in 2/26 alleles, and *IDUA* NM_00203. 4: c.1743C>G-p.(Tyr581*) in 1/26 alleles. This molecular study unveils the predominance of p.(Pro533Arg) variation in our MPS I patients. In this series, the occurrence of some clinical features linked to the Scheie syndrome is consistent with the literature, such as systematic valvulopathies, corneal opacity, and umbilical hernia; however, storage signs, facial dysmorphic features, and hepatomegaly were more frequent in our series. Screening measures for these debilitating diseases in highly consanguineous at-risk populations must be considered a priority health problem.

## 1. Introduction

Mucopolysaccharidoses (MPS’s) are a group of lysosomal genetic disorders resulting from a deficiency of acid hydrolase, which is required for the degradation of sulfated glycosaminoglycans (GAGs). These complex carbohydrates are found in both cell surfaces and extracellular matrix in all tissues. MPS’s include several subtypes according to the affected metabolic pathway and accumulated substrate. As a result of single or combined enzyme deficiency, heparan sulfate, dermatan sulfate, chondroitin sulfate, keratan sulfate, or hyaluronan catabolism may be altered. The non-degraded lysosomal GAGs may underlie cell, tissue, and organ dysfunction. GAG fragments generated by alternative pathways are excreted in urine. There are 11 known enzyme deficiencies that give rise to 7 distinct MPS’s [1].

Mucopolysaccharidosis type I (MPS I) is a rare autosomal recessive disorder caused by a deficiency of α-l-iduronidase (IDUA, EC3.2.1.76). IDUA degrades complex polysaccharides by removing a single α-l-iduronyl residue from heparan sulfate and dermatan sulfate. Partially degraded GAGs accumulate progressively and lead to multiorgan dysfunction and damage [1]. The symptoms range from the severe Hurler form (MPS IH-OMIM #607014) to the more attenuated Hurler-Scheie (MPS IH/S-OMIM #607015) and Scheie (MPS IS-OMIM #607016) phenotypes. The classification is mainly based on the age at first symptoms and the presence or not of mental retardation. The common clinical features found in the severe form of the disease consist in facial dysmorphism (86.4%), corneal clouding (70.9%), hepatomegaly (70%), dysostosis multiplex (43.6%) and hernias (58.9%). In the Hurler/Scheie syndrome, five symptoms are often described: corneal clouding (68.3%), facial dysmorphism (72.7%), hepatomegaly (66.5%), hernia (59.9%) and joint stiffness (57.3%). Since there is no typical clinical profile in Scheie syndrome, diagnosis may be challenging. Some complications may be observed such as valvulopathy (67.7%), corneal clouding (70.1%), joint stiffness (69.3%), hernia (53.5%) and carpal tunnel syndrome (51.2%). Storage symptoms are present only in about 50% of the cases, including facial dysmorphism (48%) and hepatomegaly (48%). The average survival age is of 28 years that implies patient’s shift from pediatric to adult management [2].

The *IDUA* gene is localized on chromosome 4p16.3. It spans approximately 19 kb and contains 14 exons. An intron of 566 bp separates the first two exons. A large intron of approximately 13 kb follows. The last 12 exons are clustered within 4.5 kb [3]. *IDUA* encodes a precursor protein of 653 amino acids, which is glycosylated and then processed to the mature form [4,5]. To date, 222 disease variations have been reported including missense/nonsense variations, deletion and insertion, and splicing variants (Human Gene Mutation Database-HGMD) [6]. MPS I is a rare disease with an estimated global incidence of 1:100,000 live births [2]. The birth incidence is low in different populations—The Netherlands (1.19), Germany (0.69), Australia (1.14), and the UK (1.07) [7]. However, in some high-risk populations, the lack of such data may lead to underdiagnoses.

In this study, 13 MPS I Algerian patients from 11 distinct families including 10 Scheie, 1 Hurler-Scheie, and 2 Hurler patients have been characterized at the clinical and molecular level.

## 2. Results

### 2.1. Clinical Features

A series of 13 Algerian patients from 11 different families was characterized. Patients P5 and P6 and Patients P7 and P8 are brothers and sisters. Sex ratio is 5 males:8 females. Patient ages span from 1.7 years (P7) to 22.6 years (P3). Median age is 13.2 years. The percentage of consanguineous marriage is 85%. Diagnostic median age is 8 years. Except for one patient (P7), for whom the diagnosis was done very early (at 11 months) following the confirmation of his sister’s diagnosis, all patients had been diagnosed after the age of 2.5 years. The earliest diagnosis (P12) was at 2.6 years, and the latest was at 15.7 years old (P3).

All patients were born at term; birth weight (BW) is known for 8 patients. Three were eutrophic, 4 were macrosomic (BW > 4 kg at term), and one patient presented a mild growth retardation. All patients showed facial dysmorphic features. Macrocrania is found in 7 patients (54%), macroglossia in 3 patients (23%), and an organomegaly in 10 patients (77%). Umbilical hernia is found in all patients with a median detection at 5.5 years. Delayed growth is found in 10 out of 12 documented patients. All these patients showed low height. Two patients (P12 and P13) presented with a typical Hurler symptomatology. Patient P12 died at 9 years old due to respiratory failure. The death age is relatively high compared to the survival median reported in different studies (3.8 years in the GIR). Patient P13 presented with mental retardation, respiratory failure and hydrocephalus which required neurosurgery. Facial dysmorphic features were detected early, at the age of 14 months. Eleven of the 13 patients presented with a mild form (1/11: MPS IS/H with mild mental retardation and 10/11: MPS IS without mental retardation). Main clinical features are presented in Table 1.

### 2.2. Molecular Analyses

The amplification and the sequencing of the genomic DNA of 14 exons of *IDUA* gene including promoter region and intron-exon junctions were performed. Molecular analysis revealed four pathological variations (Table 1) at the homozygous or heterozygous composite state: *IDUA* NM_00203.4:c.1598C>G-p.(Pro533Arg) in 21/26 alleles, *IDUA* NM_00203.4:c.532G>A-p.(Glu178Lys) in 2/26 alleles, *IDUA* NM_00203.4:c.501C>G-p.(Tyr167*) in 2/26 alleles, and *IDUA* NM_00203.4: c.1743C>G-p.(Tyr581*) in 1/26 alleles. The c.1598C>G-p.(Pro533Arg) variation was found at the homozygous state in 10 patients (P1–P10). Patient P11 was heterozygote for this variation and a second heterozygote variation, located in exon 13, c.1743C>G-p.(Tyr581*), has been characterized. Patients P11 and P13 were homozygous for a missense variation c.532G>A-p.(Glu178Lys) and a nonsense variation c.501C>G-p.(Tyr167*), respectively.

## 3. Discussion

In this study, we report on the clinical characteristics, biochemical, and molecular profiling of a series of 13 MPS I Algerian patients. Most of the studied patients presented a mild type (84%). Indeed, the patients have been classified as following: 10 MPS IS with preserved mental development, 1 MPS IH/S with mild cognitive impairment, and 2 MPS IH with severe mental retardation. It is worth noting that most MPS I studies in different populations report higher frequencies of severe phenotypes [2] (88% in Germany [7], 54% in Taiwan [8]). In addition, 57% of included patients in the Genzyme International Registry (GIR) have a severe phenotype [9]. Other studies on North African populations also showed a high proportion of severe phenotypes, 62% in Morocco [10], and 70% in Tunisia [11,12,13]. However, recent studies reported a relatively low percentage of severe forms in China [2,14]. The lower proportion of severe forms in our study may be related to (i) the absence of specialized centers in Algeria; (ii) the lack of awareness and health disparities in the country; and (iii) the high early mortality among the subjects with severe phenotypes prior to any investigation. This selection bias favors the delayed diagnosis of mild phenotypes.

Phenotypic variability is a frequent phenomenon in lysosomal storage diseases. In this series, no correlation was observed between the clinical severity and either the urinary GAG concentration or the residual enzyme activity (Table 1). It is likely that other genetic and epigenetic factors underlie the clinical variability, particularly in patients with late onset disease.

The large variability of the mutational profile of the *IDUA* gene is considered a major factor of the clinical heterogeneity of MPS I. Extensive work have been done to search for genotype–phenotype correlation in MPS I patients [15]. In this study, we have studied for the first time, the mutational profile of the *IDUA* gene in an Algerian series of 13 MPS I patients. The recruitment focused specifically on Western Algeria (Oran Hospital). Two missense variations (c.1598C>G-p.(Pro533Arg) and c.532G>A-p.(Glu178Lys)) and two nonsense variations (c.501C>G-p.(Tyr167*) and c.1743C>G-p.(Tyr581)) have been identified. The p.(Pro533Arg) variation is clearly predominant in our series (21/26 studied alleles). It leads to the substitution of a neutral amino acid (Proline) by a basic amino acid (Arginine); the produced protein is misfolded but still functional [16]. A large clinical variability in patients carrying this variation has been described [17]. This variant was identified for the first time by Scott *et al.* in 1992 in patient with severe form [18]. In our series, ten patients presented with homozygous p.(Pro533Arg). Nine of them have been classified as a Scheie phenotype, while patient P10 had a more severe clinical picture with mild mental retardation. This clinical presentation illustrates that metabolic diseases are more than simple mendelian diseases and a putative modifier gene could modulate their phenotypes. The 84% proportion of this sequence variation in our series is consistent with Chkioua’s results in Tunisia (64%) [11,12,13] and Alif’s results from Morocco (92%) [10]. The origin of this variation may be Mediterranean, with a probable founding effect in Sicilia where it is identified in 11% of patients [10]. The high amount of consanguineous marriages in North Africa (around 32% in Tunisia and 22.6% in Algeria) associated with a large family size could favor the expression of autosomal recessive diseases. This consanguinity rate which reaches 60% in rural regions [12] may explain the high proportion of the variation p.(Pro533Arg) in this population. In our study, the consanguinity rate reaches 85%. In addition, these patients are from the same region of Algeria (Western Algeria), which may constitute a selection bias and could also explain the high proportion of p.(Pro533Arg) variation. The second missense variant found in this study is p.(Glu178Lys) identified in Patient P11 at a homozygous state. This variant has been previously described in three patients: together with the deletion c:134-145del12–p.(Phe46_Ser49del) in an Italian patient [19], with a nonsense variation c:1205G<A-p.(Trp402*) in another Italian patient [20], and in a Chinese patient in whom the second variant was not identified [14]. These three patients presented with an intermediate phenotype (MPSI H/S). Thus, this variation seems to have an attenuating effect since the two variations p.(Phe46_Ser49del) and p.(Trp402*) have been associated with a severe phenotype when found in a homozygous state [19,21]. The tow nonsense variations identified in this study were associated with a Hurler phenotype. Patient P11 carries the variations p.(Pro533Arg) and p.(Tyr581*). The presence of the nonsense variant probably worsens the phenotype. The second nonsense variation p.(Tyr167*) was identified at the homozygous state in Patient P13. This variation has been described at the heterozygous state in only two patients, once in an attenuated phenotype associated with a missense variation c:613T>A-p.(Cys205Tyr) [22], then in a severe phenotype carrying another nonsense variation c:208C>T-p.(Gln70*) [23]. As long as there is no established severity scale for the clinical assessment of MPS I patients, inter-cohort comparison studies and genotype–phenotype correlation is needed.

## 4. Material and Methods

### 4.1. Patients

We report on the phenotypes and molecular characteristics of 13 Algerian patients from 11 distinct families presenting with MPS I. All patients included in this study were seen at the outpatient clinic by a pediatrician. Cognitive ability was assessed via clinical observation. A questionnaire, requesting information on pregnancy, first clinical signs, mental and motor milestones, behavioral problems, sleeping problems, and medical history, was filled out by caregivers. Written informed consents were obtained from the parents for molecular analysis and participation to the study.

### 4.2. Clinical and Biochemical Diagnoses

Clinical findings were consistent with MPS I diagnosis. Biological diagnosis was oriented by quantitation and electrophoresis of urinary glycosaminoglycans. Enzyme activity was assayed using fluorogenic substrates revealing low or non-detectable IDUA [24].

### 4.3. Molecular Study

Genomic DNA was extracted from venous blood using QIAamp DNA Blood Mini Kit^®^ Qiagen and was amplified *in vitro* by PCR. Multiple pairs of primers were synthesized to amplify each of *IDUA* exonic regions, including intron/exon boundaries and promoters (primers sequences are available upon request). Primers used to amplify the genomic sequences were designed according to the sequence NM_000203.4. PCR reactions were carried out in 1× Thermo Scientific Buffer IV (75 mM Tris-HCL pH 8.8, 20 mM (NH_4_)_2_SO_4_, 0.01% Tween 20), 1.5 mM MgCl_2_, 100 μM dNTPs, 0.025 U/μL *taq* polymerase (Thermo Scientific, Illkirch, France), 0.6 μM of each primer. Touchdown PCR consisted of one cycle of 95 °C for 5 min for the initial denaturation step followed by 12 cycles of denaturation at 95 °C for 25 s, varying annealing (60–48 °C) for 25 s, and extension at 72 °C. Then, 35 cycles were performed as follows: denaturation at 95 °C for 25 s, annealing at 48 °C for 25 s, and extension at 72 °C for 25 s. PCR was terminated after a final cycle at 72 °C for 5 min. Direct DNA fragments sequencing was performed with an ABI prism big dye Terminator cycle Sequencing Ready Reaction Kit and ABI model 3130xl Genetic Analyser (Thermo Scientific, Illkirch, France). Patients’ genomic sequences comparison with the reference sequence is done by Variant Reporter software (Thermo Scientific, Illkirch, France). The identified variations are mined by ALAMUT software (Interactive-Biosoftware, Rouen, France). The described variations are named following the current nomenclature recommendations [25].

## 5. Conclusions

We report here on a descriptive study of a series of 13 MPS I Algerian patients presented mainly with an attenuated phenotype. Molecular studies unveiled the predominance of p.(Pro533Arg) variation. This result is consistent with similar studies performed in North African populations [10,11,12,13]. Considering the high proportion of consanguineous marriages among these populations, the incidence of such diseases is probably higher than the global estimated one. Thus, the better knowledge and the screening for inborn errors of metabolism in these countries are essential to facilitate diagnosis, patient management, genetic counseling, and prenatal diagnosis.

## Figures and Tables

**Table 1:** Clinical and molecular characteristics of the patients.

Family	F1	F2	F3	F4	F5	F5	F6	F6	F7	F8	F9	F10	F11
Patient	P1	P2	P3	P4	P5	P6	P7	P8	P9	P10	P11	P12	P13
Age (Years)	13.8	10.7	22.6	16.1	4.2	12.6	1.7	4.5	14.9	15.8	15.7	9.6	5.1
Sex	M	F	F	M	M	F	M	F	F	F	F	M	F
Consanguinity	1	1	1	1	1	1	1	1	0	1	1	0	1
Phenotype	IS	IS	IS	IS	IS	IS	IS	IS	IS	IH/S	IS	IH	IH
Genotype	p.(Pro533Arg) p.(Pro533Arg)	p.(Pro533Arg) p.(Pro533Arg)	p.(Pro533Arg) p.(Pro533Arg)	p.(Pro533Arg) p.(Pro533Arg)	p.(Pro533Arg) p.(Pro533Arg)	p.(Pro533Arg) p.(Pro533Arg)	p.(Pro533Arg) p.(Pro533Arg)	p.(Pro533Arg) p.(Pro533Arg)	p.(Pro533Arg) p.(Pro533Arg)	p.(Pro533Arg) p.(Pro533Arg)	p.(Glu178Lys) p.(Glu178Lys)	p.(Pro533Arg) p.(Tyr581*)	p.(Tyr167*) p.(Tyr167*)
GAG quantitation (fold change to the reference range mean according to age)	7	47	95	18	11	22	/	44	23	46	10	/	19.6
IDUA Enzyme activity (% to the reference range mean)	0	0	6	7	15	10	9	10	0	0	5	0	2
Diagnostic age (Years)	5.5	8.7	15.7	13	3.1	11.4	0.9	2.7	8.3	8	15.5	2.6	2.7
Weight (Kg)	28	21	27	38	17	24	11	15	35	31	33	/	18
(SD)	(−2.5)	(−2.5)	(−3)	(<−3)		(−3)	(−1)	(−1)	(−3)	(<−3)	(−3)		
Height (cm)	132	122	128	146	93	121	78	90	135	131	142	/	96
(SD)	(−3.5)	(−3)	(<−4)	(<−4)	(−3)	(<−4)	(−1.5)	(−3.5)	(<−4 )	(<−4)	(−3.5)		(−2.5)
Mental retardation	0	0	0	0	0	0	0	0	0	1	0	1	1
Facial dysmorphic features	1	1	1	1	1	1	1	1	1	1	1	1	1
Multiple dysostosis	1	1	1	1	/	1	0	0	1	1	1	1	1
Joint stiffness	1	0	1	1	0	1	0	0	0	1	1	1	1
HM/HSM	HM	0	0	HM	HM	HSM	HM	HSM	HSM	HSM	0	HSM	HM
Hernia	OM	OM	OM	OM	OM	OM	OM	OM	OM	OM	OM	OM	OM
Chronic rhinorrhea	1	1	1	0	1	1	1	1	0	1	0	1	1
Deafness	BT	BT	/	/	/	/	/	/	Mixed	P	Mixed	/	/
Corneal opacity	0	1	1	1	1	1	1	1	0	1	1	0	1
Cardiac manifestations	MI	MI + AI	MI + AI	MS + AS	MI	MS + AS	MI + AI	MI	MI + AI	MS	MI	/	0
Hydrocephalus	1	1	1	0	/	/	0	0	1	1	0	1	1

1: Present; 0: Absent; M: Male; F: Female; IH: Hurler (Severe); IH/S: Hurler-Scheie; IS: Scheie; SD: Standard Deviation; OM: Ombelical hernia; HM: Hepatomegaly; HSM: Hepatosplenomegaly; BTD: Bilateral transmission deafness; Mixed: Mixed deafness; P: Perceptive deafness; MI: Mitral Insufficiency; MS: Mitral Stenosis; AI: Aortic Insufficiency; AS: Aortic Stenosis; /: No data.

## Abbreviations

BW	Birth weight
GAGs	glycoaminoglycanes
HGMD	Human Gene Mutation Database
MPS	Mucopolysaccharidosis
OMIM	Online Mendelian Inheritance in Man

## References

[1] Neufel E.F., Muenzer J., Scrive C., Beaudet A.L., Sly W., Vaele D. (2001). The mucopolysaccharidoses. The Metabolic and Molecular Basis of Inherited Disease.

[2] Beck M., Arn P., Giugliani R., Muenzer J., Okuyama T., Taylor J., Fallet S. (2014). The natural history of mps i: Global perspectives from the mps i registry. Genet. Med..

[3] Scott H.S., Guo X.H., Hopwood J.J., Morris C.P. (1992). Structure and sequence of the human α-l-iduronidase gene. Genomics.

[4] Brooks D.A. (1993). Review: The immunochemical analysis of enzyme from mucopolysaccharidoses patients. J. Inherit. Metab. Dis..

[5] Taylor J.A., Gibson G.J., Brooks D.A., Hopwood J.J. (1991). α-l-Iduronidase in normal and mucopolysaccharidosis-type-I human skin fibroblasts. Biochem. J..

[6] Human gene mutation database. http://www.hgmd.cf.ac.uk/ac/index.php.

[7] Moore D., Connock M., Wraith E., Lavery C. (2008). The prevalence of and survival in Mucopolysaccharidosis I: Hurler, Hurler-Scheie and Scheie syndromes in the UK. Orphanet J. Rare Dis..

[8] Lin H.Y., Lin S.P., Chuang C.K., Niu D.M., Chen M.R., Tsai F.J., Chao M.C., Chiu P.C., Lin S.J., Tsai L.P. (2009). Incidence of the mucopolysaccharidoses in Taiwan, 1984–2004. Am. J. Med. Genet. A.

[9] D’Aco K., Underhill L., Rangachari L., Arn P., Cox G., Giugliani R., Okuyama T., Wijburg F., Kaplan P. (2012). Diagnosis and treatment trends in mucopolysaccharidosis I: Findings from the MPS I Registry. Eur. J. Pediatr..

[10] Alif N., Hess K., Straczek J., Sebbar S., N’Bou A., Nabet P., Dousset B. (1999). Mucopolysaccharidosis type I: Characterization of a common mutation that causes Hurler syndrome in Moroccan subjects. Ann. Hum. Genet..

[11] Chkioua L., Khedhiri S., Kassab A., Bibi A., Ferchichi S., Froissart R., Vianey-Saban C., Laradi S., Miled A. (2011). Molecular analysis of mucopolysaccharidosis type I in Tunisia: Identification of novel mutation and eight Novel polymorphisms. Diagn. Pathol..

[12] Chkioua L., Khedhiri S., Jaidane Z., Ferchichi S., Habib S., Froissart R., Bonnet V., Chaabouni M., Dandana A., Jrad T. (2007). Mucopolysaccharidosis type I: Identification of alpha-l-iduronidase mutations in Tunisian families. Arch. Pediatr..

[13] Chkioua L., Khedhiri S., Turkia H.B., Tcheng R., Froissart R., Chahed H., Ferchichi S., Ben Dridi M.F., Vianey-Saban C., Laradi S. (2011). Mucopolysaccharidosis type I: Molecular characteristics of two novel alpha-l-iduronidase mutations in Tunisian patients. Diagn. Pathol..

[14] Wang X., Zhang W., Shi H., Qiu Z., Meng Y., Yao F., Wei M. (2012). Mucopolysaccharidosis I mutations in Chinese patients: Identification of 27 novel mutations and 6 cases involving prenatal diagnosis. Clin. Genet..

[15] Thomas J.A., Beck M., Clarke J.T., Cox G.F. (2010). Childhood onset of Scheie syndrome, the attenuated form of mucopolysaccharidosis I. J. Inherit. Metab. Dis..

[16] Matte U., Yogalingam G., Brooks D., Leistner S., Schwartz I., Lima L., Norato D.Y., Brum J.M., Beesley C., Winchester B. (2003). Identification and characterization of 13 new mutations in mucopolysaccharidosis type I patients. Mol. Genet. Metab..

[17] Terlato N.J., Cox G.F. (2003). Can mucopolysaccharidosis type I disease severity be predicted based on a patient’s genotype? A comprehensive review of the literature. Genet. Med..

[18] Scott H.S., Litjens T., Nelson P.V., Brooks D.A., Hopwood J.J., Morris C.P. (1992). α-l-Iduronidase mutations (Q70X and P533R) associate with a severe Hurler phenotype. Hum. Mutat..

[19] Venturi N., Rovelli A., Parini R., Menni F., Brambillasca F., Bertagnolio F., Uziel G., Gatti R., Filocamo M., Donati M.A. (2002). Molecular analysis of 30 mucopolysaccharidosis type I patients: Evaluation of the mutational spectrum in Italian population and identification of 13 novel mutations. Hum. Mutat..

[20] Bertola F., Filocamo M., Casati G., Mort M., Rosano C., Tylki-Szymanska A., Tuysuz B., Gabrielli O., Grossi S., Scarpa M. (2011). IDUA mutational profiling of a cohort of 102 European patients with mucopolysaccharidosis type I: Identification and characterization of 35 novel α-l-iduronidase (IDUA) alleles. Hum. Mutat..

[21] Scott H.S., Litjens T., Hopwood J.J., Morris C.P. (1992). A common mutation for mucopolysaccharidosis type I associated with a severe Hurler syndrome phenotype. Hum. Mutat..

[22] Beesley C.E., Meaney C.A., Greenland G., Adams V., Vellodi A., Young E.P., Winchester B.G. (2001). Mutational analysis of 85 mucopolysaccharidosis type I families: Frequency of known mutations, identification of 17 novel mutations and *in vitro* expression of missense mutations. Hum. Genet..

[23] Vazna A., Beesley C., Berna L., Stolnaja L., Myskova H., Bouckova M., Vlaskova H., Poupetova H., Zeman J., Magner M. (2009). Mucopolysaccharidosis type I in 21 Czech and Slovak patients: Mutation analysis suggests a functional importance of C-terminus of the IDUA protein. Am. J. Med. Genet. A.

[24] Whiteman P., Henderson H. (1977). A method for the determination of amniotic-fluid glycosaminoglycans and its application to the prenatal diagnosis of Hurler and Sanfilippo diseases. Clin. Chim. Acta.

[25] Human genome variation society. http://www.hgvs.org/mutnomen.

